# Volatile Composition of Industrially Fermented Table Olives from Greece

**DOI:** 10.3390/foods10051000

**Published:** 2021-05-02

**Authors:** Theano Mikrou, Katerina Kasimati, Ioanna Doufexi, Maria Kapsokefalou, Chrysavgi Gardeli, Athanasios Mallouchos

**Affiliations:** Laboratory of Food Chemistry and Analysis, Department of Food Science and Human Nutrition, Agricultural University of Athens, Iera Odos 75, 118 55 Athens, Greece; thmikrou@aua.gr (T.M.); kskaterina96@gmail.com (K.K.); iwannakidf1@gmail.com (I.D.); kapsok@aua.gr (M.K.); agardeli@aua.gr (C.G.)

**Keywords:** table olives, volatiles, SPME, Conservolea olives, Halkidiki olives, Kalamata olives, Greek-style fermentation, Spanish-style fermentation, GC-MS

## Abstract

Table olives represent one of the most important fermented products in Greece. Their highly appreciated flavor is directly associated with the volatile composition. However, extensive data on the volatile profile of table olives from Greek cultivars are scarce in the literature. For this reason, the volatile components of industrially fermented table olives from Kalamata, Conservolea and Halkidiki cultivars grown in different geographical areas within Greece were determined using headspace solid-phase microextraction combined with gas chromatography–mass spectrometry. More than 100 volatile compounds were identified and distributed over different chemical classes. All samples were rich in esters, alcohols and acids, whereas the samples of cv. Halkidiki were also characterized by increased levels of volatile phenols. Both qualitative and quantitative differences were observed, which resulted in the discrimination of the table olives according to olive cultivar and growing location. To the best of our knowledge, this is the first systematic study on the volatile profiles of table olives from Greek cultivars that also highlights the pronounced effect of olives’ growing location.

## 1. Introduction

Table olives are currently the most popular fermented vegetable product in Western countries and constitute, along with olive oil, an important ingredient of the Mediterranean diet. Worldwide production for the 2019/2020 season was around 3.06 million tons, of which 25.1% was produced in the European Union, predominantly in Spain (458,000 t), Greece (222,000 t) and Italy (60,000 t) [1]. The three most economically important cultivars (cv.) grown in Greece for table olive processing are Conservolea, Kalamata and Halkidiki, which are found as finished products under the brand names Conservolea (or Amfissis) and Kalamata (or Kalamon) natural black olives as well as Halkidiki green olives [2,3]. 

The cv. Conservolea is primarily grown in central Greece (including the prefectures of Fthiotida, Magnesia and Evia) and can be processed using either the Spanish-style or Greek-style method, although the latter is preferred. Likewise, natural black olives are produced from cv. Kalamata, which is cultivated mainly in western Greece (prefecture of Aitoloakarnania) and southern Peloponnese (Messinia, Lakonia) [2]. During Greek-style processing, the olives are harvested when they are fully ripe or just before full ripeness, immersed directly in a NaCl solution (6–10% *m*/*v*) and subjected to spontaneous fermentation for 8–12 months, mainly promoted by indigenous yeasts and lactic acid bacteria (LAB) [4]. 

The cv. Halkidiki is grown in northern Greece, primarily in the prefecture of Halkidiki as well as in Kavala. After harvesting, the green olives are processed using the Spanish-style method by treating the fruit with a lye solution (NaOH 2–3% *m*/*v*) to hydrolyze the bitter glucoside oleuropein. After a washing step to remove the excess alkali, a NaCl solution (8–12% *m*/*v*) is added, and fermentation takes place promoted mainly by LAB, which lasts for 3–7 months [5].

The primary purpose of table olive fermentation is to achieve a preservation effect and enhance the organoleptic properties of the final product [6]. The pleasant fruity flavor of table olives, a highly appreciated characteristic from consumers, is closely associated with the composition of volatile compounds, which depends on various factors, including olive cultivar, fruit ripening degree, climatic and processing conditions [7]. Earlier works were carried out to examine mostly the major volatiles in the brines of industrially fermented green olives of Spanish cultivars [8], to screen for potent odorants in Moroccan green olives [9], to compare the inoculated versus the spontaneous fermentation of Conservolea cv. green [6] and black olives [10], Halkidiki green olives [11], Moresca and Kalamata cv. Greek-style olives [12], and to compare the volatile compounds in Spanish-style, Greek-style and Castelvetrano-style green olives of Nocellara del Belice cv. [13]. 

During the last decade, the powerful technique of solid-phase microextraction (SPME) in combination with gas chromatography–mass spectrometry (GC-MS) has been applied by several researchers to study in more depth the volatile composition of table olives. A total of 42 compounds were identified in *alcaparras* stoned table olives produced from Portuguese olive cultivars (cv. Cobrancosa, Madural, Negrinha de Freixo, Santulhana and Verdeal Transmontana). Aldehydes were the major chemical class in all cultivars studied, with the main contributors being hexanal, phenylacetaldehyde and (E,E)-2,4-heptadienal. By using principal component analysis (PCA) the olive cultivars were distinguished based on their volatile profile [14]. In a similar manner, Aponte et al. [15] demonstrated that the volatile profile of green Sicilian table olives was dependent on the cultivar as well as the storage period in brine. As regards black-ripe (or Californian-style) table olives, the effects of olive cultivar (Manzanillla, Hojiblanca), growing area and storage period on the volatile composition have been recently disclosed [16,17,18]. A total of 74 volatile compounds were identified in the headspace of samples, of which 12 were identified as significant volatiles contributing to the discrimination between Manzanilla and Hojiblanca black ripe olives [17]. A few studies have examined the influence of starter cultures on the volatile profile of Greek-style processed green [19,20,21,22] and black [23] table olives from Italian cultivars (Giarraffa, Grossa di Spagna, Nocellara del Belice, Bella di Cerignola, Nocellara Etnea). In addition, several works have been carried out on Spanish-style processed table olives produced from Spanish cultivars (Manzanilla, Gordal, Hojiblanca) studying their volatiles [24,25,26,27,28,29]. Only a few of them [24,30] have focused on the discrimination of cultivars as well as the growing areas based on volatiles. Significant differences in the proportions of volatile compounds between samples from the Gordal cv. and those from Manzanilla and Hojiblanca cv. were detected and statistically visualized by PCA. The locations where the fruits were grown for a given cultivar were not clearly distinguished, indicating that the fruit growing environment had a minor influence on the volatile composition of Spanish-style green table olives [30]. 

However, extensive data on volatile composition of table olives from Greek cultivars are scarce in the literature. Bleve et al. [31] studied Greek-style fermentations of Kalamata and Conservolea cv. black olives and monitored, among other parameters, the evolution of volatiles to identify useful chemical markers for the fermentation process. Chytiri et al. [32] studied the effect of different inoculation strategies of selected starter cultures on fermentations using the above-mentioned Greek varieties. To our knowledge, no work has been reported so far for the volatile profile of Halkidiki green tables. 

The main objective of the present work was to assess the volatile profile of (a) Greek-style fermented black olives of Conservolea and Kalamata cultivars, and (b) Spanish-style fermented green olives of cv. Halkidiki, which constitute the most prominent table olives commercially produced in Greece. The samples were fermented on the industrial scale and originated from different geographical regions for each olive cultivar. Using a volatilomics approach, the study aims to identify useful volatile chemical markers for the discrimination of the samples according to cultivar and growing location. This will aid to develop a reliable method for authentication purposes since consumers show a greater preference for products whose varietal or/and geographical origin is declared.

## 2. Materials and Methods 

### 2.1. Samples

A total of 59 fermented table olive samples—29 samples of cv. Kalamata natural black olives, 15 samples of cv. Conservolea natural black olives and 15 samples of cv. Halkidiki green olives—were obtained during the 2018–2019 harvesting period directly from local table olive producing companies. Specifically, the drupes of cv. Kalamata were collected from two geographical regions, namely Aitoloakarnania and Southern Peloponnese (Lakonia, Messinia) at the right stage of ripening (i.e., 3/4 of the mesocarp had attained black color) and processed in the company’s facilities using the Greek-style method. Spontaneous fermentation was undertaken in 5000–6000 L capacity polyester vessels containing 60–70% olives and 30–40% brine (NaCl 5–6% *m*/*v*). The same processing method was applied for the drupes of cv. Conservolea, which had been collected from Fthiotida, Magnesia and northern Evia growing areas. The drupes of cv. Halkidiki were collected from Halkidiki and Kavala regions and processed using the Spanish-style method. The olives were initially subjected to a washing step with tap water to remove any impurities and subsequently immersed in a 1.9% (*m*/*v*) NaOH solution for 10–12 h at room temperature (20–22 °C) until the alkali penetrated approximately 2/3 of the flesh as measured from the epidermis to the pit. A washing step was followed, replacing the NaOH solution with tap water. The process included two water changes at 4 and 8 h to remove the residual lye from the olive flesh. Spontaneous fermentation was undertaken in 6000 L capacity polyester vessels using a 10% (*m*/*v*) NaCl solution (brine/olive ratio 1.5/1). At the onset of fermentation, the brines were acidified with 0.1% (*v*/*v*) lactic acid and 0.01% (*v*/*v*) HCl solution. 

All samples (3 kg each) were taken in coincidence with the final stage of fermentation (8 months for Greek-style and 4 months for Spanish-style processed olives), transferred to the laboratory in plastic vessels filled with brine and analyzed within two weeks. The sample coding and further details are presented in Table 1. Figure S1 presents the map of olives’ growing areas. Physicochemical, microbiological and sensory analyses of the samples have been described elsewhere [2,3]. 

### 2.2. Headspace Solid-Phase Microextraction (SPME)

Approximately 50 g of each olive sample was destoned manually and snap-frozen in liquid nitrogen. Tissue grinding was performed in a pre-cooled A11 analytical mill (IKA, Wilmington, NC, USA) to obtain a fine frozen powder. The mill was operated in pulse mode for 20–30 s to prevent the thawing of the sample. Aliquots (2 g) of each powdered sample were accurately weighed (±0.1 mg) into 10 mL glass vials and sealed with a polypropylene cap with PTFE/Silicon septum. Subsequently, the vials were placed in a water bath at 40 °C for 15 min. Then, the SPME fiber (DVB/CAR/PDMS, 2 cm; Sigma Aldrich, Germany) was exposed to the headspace for 30 min to isolate the volatiles. Afterwards, the fiber was inserted into the injection port of the gas chromatography–mass spectrometry system (GC-MS) for 5 min at 240 °C in split mode (split ratio 1/5) to desorb the volatiles. After each sample’s analysis, the fiber was further conditioned for 5 min in another GC injection port for the removal of any volatile residues.

### 2.3. Gas Chromatography–Mass Spectrometry 

The volatiles were determined in a GCMS QP-2010 Ultra (Shimadzu Inc., Japan) instrument equipped with a suitable liner (0.7 mm i.d.; Sigma Aldrich) for SPME. The separation of compounds was accomplished in a DB-Wax capillary column (30 m, 0.25 mm i.d., 0.25 μm film thickness; Agilent, USA) with helium as a carrier gas at a constant linear velocity (36 cm/s). The oven temperature program was set initially at 40 °C for 5 min, increased at 5 °C/min to 180 °C, and then by 30 °C/min to 240 °C (held for 5 min). The MS was operated in the electron ionization mode (70 eV) with a scan range of 40–300 *m*/*z*. Source and interface temperatures were set at 200 °C and 240 °C, respectively. 

Identification of the compounds was accomplished by comparing the (i) retention indices (RIs) based on the homologous series of C8-C24 n-alkanes with those of authentic compounds (when available) and those of NIST14 library (NIST, USA); (ii) MS data with those of reference compounds and MS data obtained from NIST14. All volatile compounds used as reference standards were purchased from Sigma-Aldrich (Germany). GCMS Solution (ver. 4.30; Shimadzu), AMDIS (ver. 2.72; NIST) and NIST MS Search (ver. 2.2; NIST) software were used in the identification process. The reliability of identification (RID) was set at 3 levels. A-level: agreement of RI and MS spectra with those of an authentic compound analyzed under identical experimental conditions; B-level: agreement of RI (ΔRI < 20) and MS (match > 900); C-level: at least MS similarity match > 800. The GC peak area of each compound was obtained from the extracted mass chromatogram by selecting specific target ions (Table S1). The content of volatile compounds was expressed as normalized peak area %. 

### 2.4. Statistical Analysis

Before any processing, missing values were replaced by the 1/5 of the minimum value of each variable (volatile compound), and subsequently the data were normalized by sum (row-wise), glog-transformed and pareto-scaled (column-wise). Univariate and multivariate testing was accomplished using the MetaboAnalyst 4.0 web-based tool suite [33,34] and with Unscrambler X software (CAMO Software AS., Oslo, Norway) to identify significant variables that could differentiate the table olives according to cultivar and growing location.

## 3. Results and Discussion

### 3.1. Volatile Composition of Table Olives

In total, 126 volatile compounds were identified in the three types of table olives used. These comprised 11 acids, 30 alcohols, 50 esters, 10 carbonyls, 4 hydrocarbons, 6 phenols, 8 terpenoids and 7 miscellaneous compounds (Table 2). It is noteworthy that the volatile profile of Kalamata (KLM) table olives was characterized by a smaller number of compounds as compared to Halkidiki (HLK) and Conservolea (CNS) samples. The majority of the identified compounds (105 out of 126) have already been reported in various types of table olives, including Californian-style from Spanish cultivars [16,17,18], Spanish-style from Spanish cultivars [24,25,26,27,28,29,30,35], Greek-style from Italian cultivars [19,20,21,22,23], *alcaparras* stoned table olives from Portugal [14], Sicilian green table olives [15] and Tunisian olives [36]. However, the data are scarce in the literature as regards the volatile composition of table olives produced from Kalamata, Halkidiki and Conservolea cultivars [12,31,32]. 

**Figure 1:**
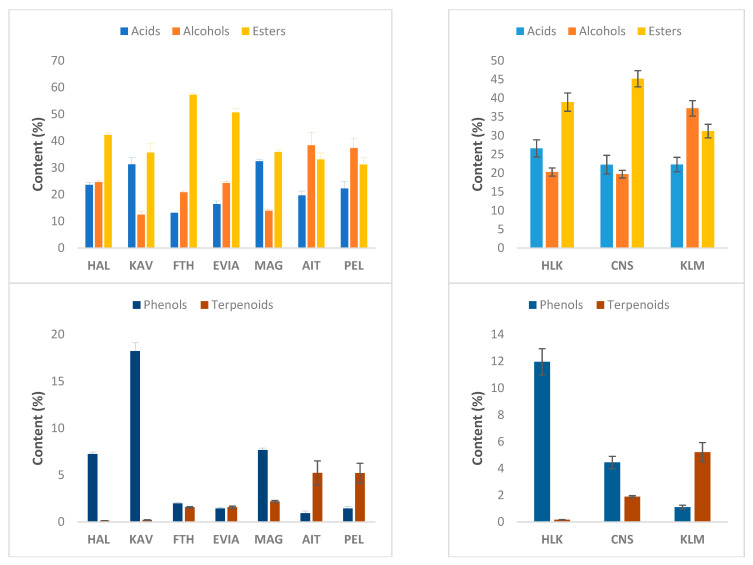

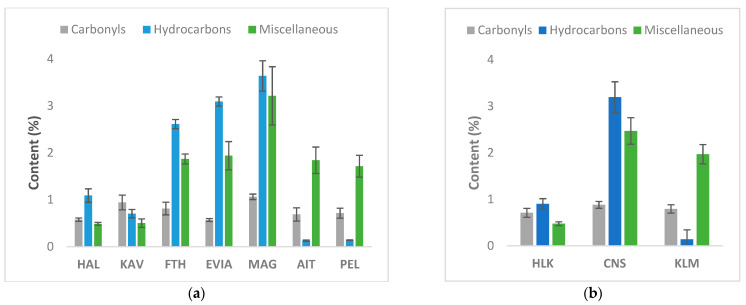
Comparison of the relative content (%) of chemical classes of the volatile compounds identified in industrially fermented table olives originating from various (**a**) growing locations (HAL: Halkidiki; KAV: Kavala; FTH: Fthiotida; EVIA: northern Evia; MAG: Magnesia; AIT: Aitoloakarnania; PEL: Southern Peloponnese) and (**b**) cultivars (HLK: Halkidiki; KLM: Kalamata; CNS: Conservolea). The error bars indicate standard errors at 95% confidence level.

The total relative content (%) of each chemical class is presented in Figure 1. The volatile profile of the three cultivars was predominated by esters (~31–45%), alcohols (~20–37%) and acids (~22–27%), whereas the content of the remaining classes was quite lower (<12%). The highest and lowest content of esters (*p* < 0.05) was observed in CNS (45.2%) and KLM (31.2%) samples, respectively. The opposite was observed for alcohol content (CNS: 19.7%, KLM: 37.3%). Acid content was similar between KLM and CNS cultivars (22.3%, *p* > 0.05), whereas a slightly higher value (*p* > 0.05) was evident in HLK samples (26.6%). However, the most prevalent differences were observed in the minor chemical classes. More specifically, the phenol content ranged from 1.1% to 11.9% for KLM and HLK samples, respectively. An opposite trend was observed for terpenoids (KLM: 5.2%, HLK: 0.17%). On the contrary, CNS samples were characterized by the highest content (*p* < 0.05) of hydrocarbons (3.2%) and miscellaneous compounds (2.5%). The sum of carbonyl compounds did not differ significantly between the cultivars. In accordance with our results, Bleve et al. [31] reported that CNS olives contained higher concentrations of esters than KLM olives. In addition, they found that alcohols were the second most abundant chemical class in CNS samples, whereas in KLM samples the alcohol content varied significantly during fermentation. In a study conducted with Spanish-style green table olives originated from three Spanish cultivars (Manzanilla, Gordal, Hojiblanca), the volatile profile was predominated by alcohols and phenols, followed by esters and acids. It is noteworthy, that the phenol content was quite high in the Spanish cultivars (~30–35%), whereas in our case (HLK samples), phenols represented ~12% of total volatiles. This could be attributed mainly to the different levels of phenolic substrates due to cultivar species or/and the degree of olive ripeness, as it will be explained later. 

As presented in Figure 1, the growing location of table olives had a significant effect on the various chemical classes within each cultivar, except for KLM. The relative amount of phenols, terpenoids and acids was higher in HLK samples produced in Kavala (KAV), whereas alcohol content was higher in samples produced in Halkidiki (HAL). Within the CNS samples, all chemical classes varied significantly between the three growing locations, namely Fthiotida (FTH), northern Evia (EVIA) and Magnesia (MAG). The latter samples contained greater amounts of phenols, acids, terpenoids, hydrocarbons, carbonyls and miscellaneous compounds (*p* < 0.05). On the other hand, esters and alcohols were significantly higher in FTH and EVIA samples, respectively. 

A closer observation of the relative content of the principal volatile compounds (Table 2) can reveal several key differences for the effect of cultivar/processing method and growing location of table olives.

Of the 11 volatile acids identified in our study, acetic acid was the dominant one followed by propanoic acid in most cases, such as HAL, FTH, EVIA, AIT and PEL samples (representing 8.7–18.1% of total volatiles). However, the dominant acids in KAV and MAG samples were propanoic acid (17.8%) and butanoic acid (19.3%), respectively. It is generally known that acetic acid is produced by bacteria such as *Acetobacter* species, *Clostridium acetobutylicum* as well as yeasts by oxidation of ethanol [13]. The formation and content of propanoic acid depends on the growth of *Propionibacterium* species [7]. Although increased levels of butanoic acid resulting from the action of *Clostridium* or *Ruminococcus* species [29] can lead to butyric spoilage, no such malodorous deterioration was observed during the organoleptic evaluation of our samples (data not shown). 

Of the 30 identified alcohols, ethanol, 2-butanol, 3- and 2-methyl-1-butanol, phenylethyl alcohol and 1-propanol were found in appreciable amounts in the samples from the three cultivars (Table 2). The highest content of ethanol (~20%) was observed in KLM samples (AIT, PEL), followed by 2-butanol and 3-methyl-1-butanol. On the contrary, 2-butanol was more abundant than ethanol in CNS samples grown in FTH and EVIA. A similar pattern was observed within the HLK table olives grown in different locations (KAV, HAL). The content of phenylethyl alcohol was higher in HLK and CNS samples (~2.0–4.0%) than KLM ones (~0.5%) and varied significantly between the growing location of each cultivar. Similar findings were observed for 1-hexanol and (*Z*)-3-hexenol. The above-mentioned results reflect different activity of yeasts and lactic acid bacteria between the cultivars as well as the growing locations of olives. It is generally known that ethanol is an end-product of the Embden–Meyerhof–Parnas glycolytic pathway, whose main function is the production of energy [13]. The biosynthesis of higher alcohols is linked to the catabolism of amino acids and sugars [37]. As suggested by Sabatini et al. [7,12], C5-C6 alcohols, aldehydes and their corresponding esters, such as 1-hexanol, 3-hexen-1-ol, hexanal and hexyl acetate, can be produced either from polyunsaturated fatty acids through the endogenous lipoxygenases or/and a similar pathway mediated by the microbial enzymes in brine. 

Esters were the most numerous chemical class of volatile compounds detected in our study. Acetate and propanoate esters were quantitatively the most important. More specifically, the dominant esters in KLM samples were mainly ethyl acetate and ethyl propanoate, followed by propyl acetate, methyl acetate, 3-methybutyl acetate and ethyl lactate (Table 2). The latter three compounds were significantly different between the two growing locations (AIT, PEL) of the KLM cultivar. On the contrary, the principal esters of CNS samples were ethyl propanoate and propyl acetate (representing ~20% of total volatiles), of which the latter was present at significantly lower levels (*p* < 0.05) in the samples from the MAG location. The same pattern was observed for propyl propanoate, which was also found in appreciable levels only in the samples from FTH and EVIA locations. It is worth to mention that several butanoate esters, such as ethyl butanoate, were detected in higher levels in MAG samples as compared to FTH and EVIA olives. This could be explained by the high content of butanoic acid in MAG olives, as mentioned before. As regards the olives of the HLK cultivar (processed with the Spanish method), the profile of esters was remarkably different between the samples grown in HAL and KAV. The most notable differences can be focused on the higher content of acetate esters, such as ethyl acetate, propyl acetate and 3-methylbutyl acetate, as well as ethyl lactate, found in HAL olives. On the other hand, the content of the major propanoate esters (methyl and propyl propanoate; ethyl propanoate excluded) was significantly higher in KAV samples. This is probably related with the corresponding higher level of propanoic acid in the latter samples, as previously mentioned. Esters are well known to contribute significantly to the flavor of both natural and fermented foods and are appreciated since they generally carry fruity flavors [38]. Acetate esters and propanoate esters could be synthesized by alcohol acyltransferase (AAT), which catalyzes the esterification of volatile alcohols with acetyl CoA and propionyl CoA, respectively, to produce volatile esters and free CoA-SH [13]. The most obvious factors that influence ester synthesis are the availability of substrates and the level of enzyme activities. Other parameters, such as pH, temperature and NaCl content, can also influence the production of esters [38].

From the six volatile phenols identified, special emphasis should be given to guaiacol and 4-methylguaiacol. They accounted for ~12% of total volatiles in HLK samples, whereas significantly lower amounts (*p* < 0.05) were observed in the CNS and KLM samples. In addition, guaiacol was found in appreciable levels only in the KAV samples as compared to HAL olives. It is known that volatile phenols are likely formed during the fermentation process as a result of microorganisms’ activity [30] via decarboxylation of phenolic acids [39]. Our results indicate that the formation of phenols can be affected primarily by cultivar and/or fermentation style and secondarily by the growing location of olives, which influences the phenolic content. Probably, the lye treatment used for the preparation of HLK samples (Spanish style) disintegrates the fruit matrix making thus the phenolic substrates more vulnerable to attack from microorganisms. This hypothesis can be strengthened by the fact that although HLK samples contained the lowest total polar phenolic content (as measured by Folin–Ciocalteu, data not shown), their content of volatile phenols was significantly higher than in Greek-style processed olives (CNS, KLM).

Among the various terpenoids identified (Table 2), trans-β-ocimene was the most abundant, characterizing solely the KLM samples from both growing locations; in the other cultivars, it was found in very low levels (<0.5%). Furthermore, the CNS samples were characterized by the presence of a-farnesene, a-muurolene and copaene. The first two compounds were not detected in the other two cultivars. Generally, terpenoids can be considered as olive-derived compounds that are released during fermentation from non-volatile precursors by the action of microbial glycosidases and by the acidic environment [25]. In fact, these sesquiterpenes have already been suggested as potential biomarkers for authenticity in table olives [30] and olive oil [40]. 

Within the class of carbonyls, most of the compounds were present at very low levels (<0.1%). The major ketone found in all samples was 2-butanone (~0.1–0.7%). Although its content was relatively higher in KAV (0.7%) and MAG (0.5%) samples, it was not statistically different (*p* > 0.05) between the cultivars studied. 3-Methylbutanal was also found in higher levels in KLM table olives. Sabatini and Marsilio [13] reported that content of 2-butanone was influenced by the processing method used in table olives of the *Nocellara del Belice* cultivar as well as by the use of inoculum in Greek-style processed Kalamata and Moresca cultivars [12]. It is noteworthy that 2-butanone has not been identified in more recent studies conducted with Greek-style processed Conservolea and Kalamata cultivars [31], Spanish-style or black-ripe processed Manzanilla, Gordal and Hojiblanca cultivars [16,25,30] and *alcaparras* table olives [14]. 

Octane was the predominant hydrocarbon in all samples, followed by 1-dodecene. Their content was significantly higher in CNS samples. Generally, alkanes and alkenes are considered secondary oxidation products of fatty acids [27,41]. It is worth noting that (E)-4,8-dimethylnona-1,3,7-triene detected here has been reported in only one study [18] as a major volatile constituent of black-ripe table olives.

### 3.2. Effect of Cultivar on Volatile Profile

**Figure 2:**
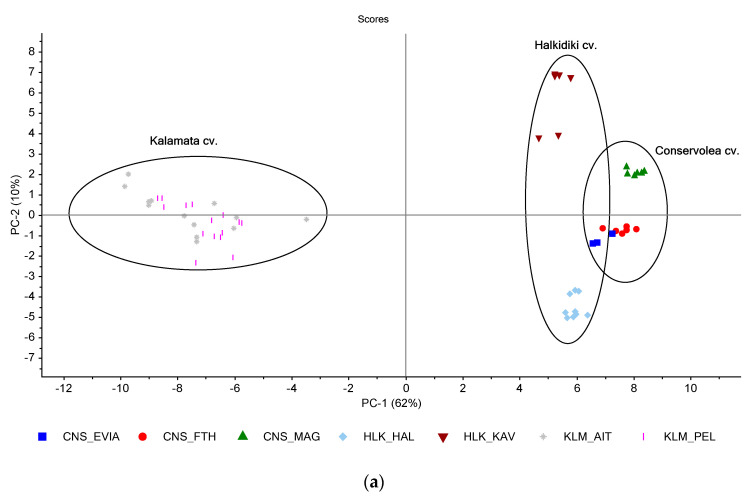

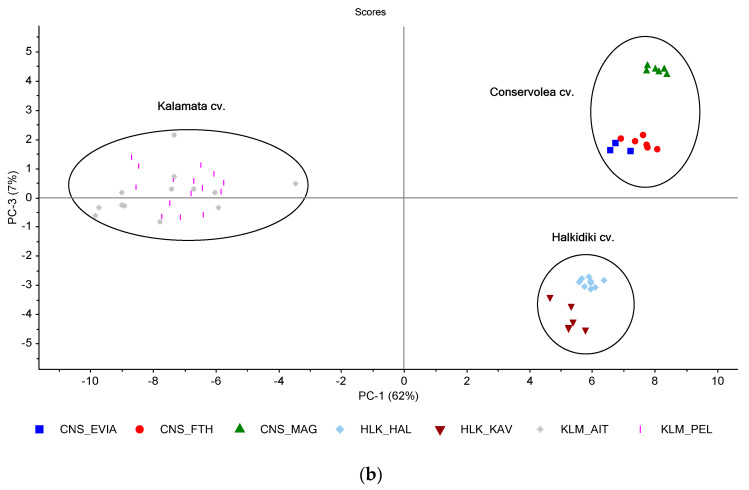
Principal component analysis (PCA) of the volatile compounds identified in industrially fermented table olives from different cultivars (CNS: Conservolea; HLK: Halkidiki; KLM: Kalamata) and growing areas (EVIA: northern Evia; FTH: Fthiotida; MAG: Magnesia; HAL: Halkidiki; KAV: Kavala; AIT: Aitoloakarnania; PEL: Southern Peloponnese): (**a**) PC1 vs PC2; (**b**) PC1 vs PC3.

It seems that the combined effect of cultivar and style of fermentation affects the volatiles’ composition. For this reason, PCA was performed using the contents of individual volatile compounds as variables (including the sum of each chemical class) to differentiate the three cultivars studied herein. The first three principal components (PCs) accounted for 79% of the total variance. KLM samples formed a cluster on the negative region of PC1, whereas CNS and HLK samples formed two major clusters on the positive region of PC1 (Figure 2a). This can be attributed to the less complex volatile profile of KLM samples. 

Within each of these clusters (CNS and HLK), two sub-clusters can be distinguished spreading in the direction of PC2, which correspond to samples of different geographical origin. Thus, PC2 describes the variation within the cultivars CNS and HLK, which can be attributed to the different growing location of table olives, as will be elaborated later. As shown in Figure 2b, PC3 spread further apart the clusters of CNS and HLK samples. Examining the corresponding loadings plots (Figure S2), it was shown that the volatile compounds mainly associated with the KLM cluster were propyl hexanoate (86), trans-β-ocimene (120), ethanol (12), 3-methylbutanal (44) as well as the class of terpenoids and alcohols (Figure S2a). 

The CNS cluster was mainly related to α-farnesene (126), α-muurolene (125), linalool (122), butyl butanoate (78), 3-methylbutyl butanoate (81), 1-dodecene (104) and Hydrocarbons class, whereas the HLK cluster was formed due to its higher content of 1,3-propanediol (39), 1-methylpropyl propanoate (64), 2-phenylethyl propanoate (101), guaiacol (113), 4-methylguaiacol (114) and the phenols class (Figure S2b). The discrimination of table olives according to cultivar was also confirmed by hierarchical cluster analysis (Figure S3). 

### 3.3. Effect of Geographical Origin on Volatile Profile

**Figure 3:**
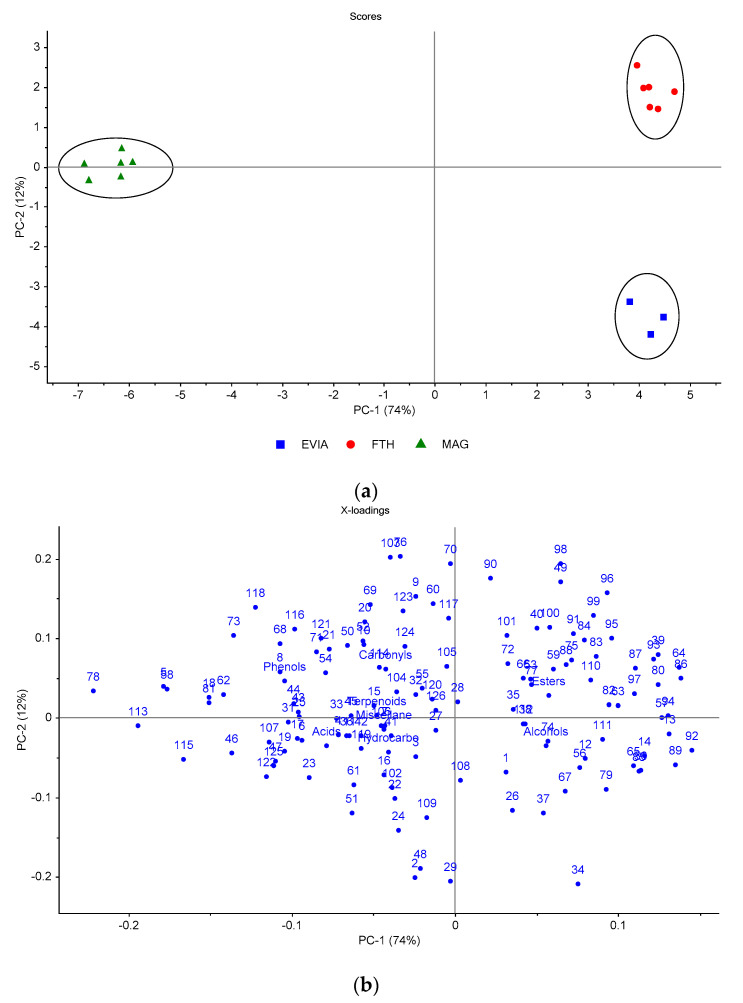
Principal component analysis of the volatile compounds identified in cv. Conservolea natural black olives grown in northern Evia (EVIA), Fthiotida (FTH) and Magnesia (MAG): (**a**) scores plot, (**b**) loadings plot of the first two principal components. The codes of variables are given in Table 2.

After these encouraging results, the samples from each cultivar were grouped according to their growing location (Table 1), and PCA was conducted to reveal any discriminating variables (volatiles). The CNS table olives were produced in Magnesia (MAG), Fthiotida (FTH) and northern Evia (EVIA). These neighboring regions are in central-eastern Greece (Figure S1). As shown in Figure 3a, three distinct clusters are evident in the plot of the two first PCs, which both accounted for 86% of the total variance.

The MAG cluster is placed on the negative side of PC1 due to its high content of butyl butanoate (78), methyl butanoate (58), ethyl butanoate (62), 3-methylbutyl butanoate (81), butanoic acid (5), butanol (18), guaiacol (113) phenol (115), acetoin (46) as well as the phenols class (Figure 3b). The other two clusters (FTH, EVIA) are both on the positive side of PC1, which seems reasonable due to the high proximity of these geographical regions (Figure S1). The volatile compounds mainly associated with the FTH cluster were the esters class as well as the individual esters benzyl acetate (96), benzyl propanoate (98), ethyl 2-hydroxybenzoate (99), methyl benzoate (95), ethyl 3-hexenoate (83), (Z)-3-hexenyl acetate (84), 1-methylpropyl propanoate (64) and ethyl hexanoate (80). On the other hand, EVIA samples were related mostly with ethanol (12), 2-butanol (13), propanol (14), (Z)-2-hexen-1-ol (30), (E)-2-hepten-1-ol (34), ethyl 2-methylbutanoate (65), ethyl 3-methyl-2-butenoate (79), methyl lactate (85), ethyl lactate (89) and isopropyl lactate (92). Similar results were observed on the heatmap (Figure S4) of the hierarchical cluster analysis. Furthermore, it is evident that PC2 incorporated the discriminating variation between the FTH and EVIA clusters because they were placed in opposite quadrants along the y-axis. This differentiation can be attributed mostly to the higher content of (E)-2-hexenol (29) and 3-methylbutyl acetate (70) in EVIA and FTH samples, respectively (Figure 3b).

**Figure 4:**
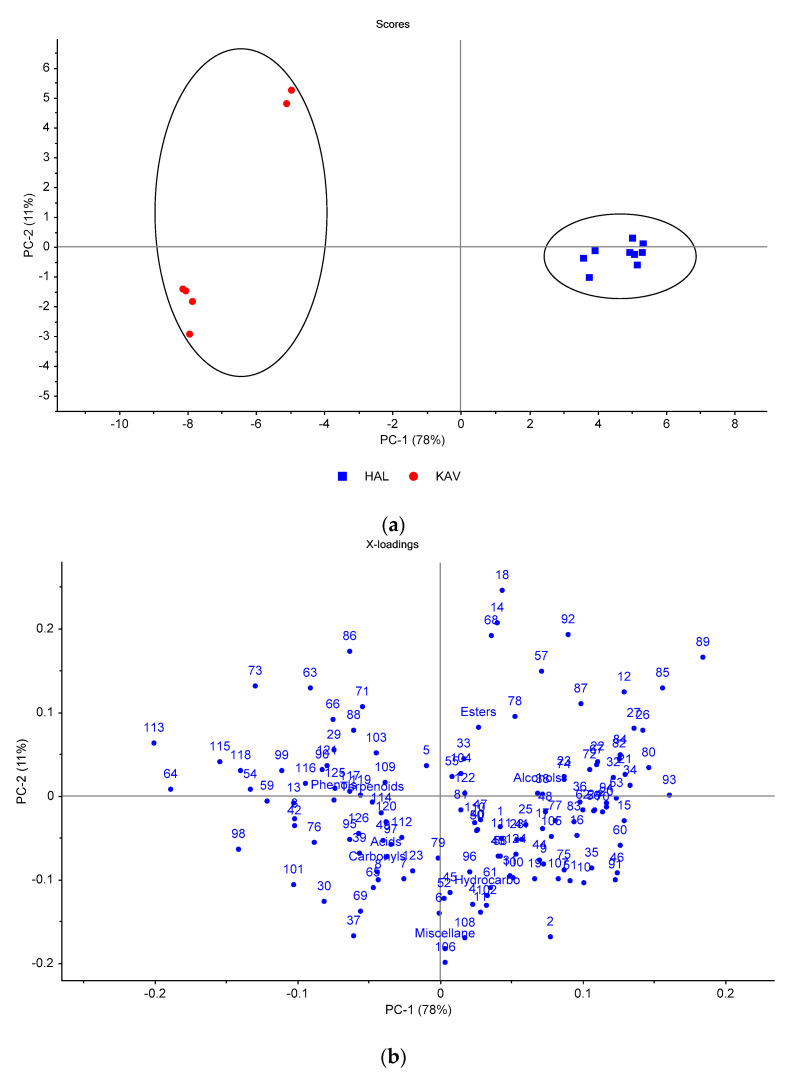
Principal component analysis of the volatile compounds identified in cv. Halkidiki green olives grown in Halkidiki (HAL) and Kavala (KAV): (**a**) scores plot, (**b**) loadings plot of the first two principal components. The codes of variables are given in Table 2.

HLK table olives were produced in Halkidiki (HAL) and Kavala (KAV) (Figure S1) using the Spanish-style method. It is evident from the PCA scores plot (Figure 4a) that the KAV samples clustered in the negative side of PC1, whereas HAL samples were on the positive side of PC1. Thus, PC1 compensates all the information (78% of explained variance) that differentiates HLK table olives according to growing location. PC2 and PC3 explain the variance within each cluster (Figure 4a, Figure S5). The examination of loading plot (Figure 4b) revealed that the KAV samples were characterized mainly by a higher content of volatile phenols (including the sum of phenols’ class), such as guaiacol (113), phenol (115) and 4-methylphenol (118). In addition, they were richer in propanoate esters, such as 1-methylpropyl (64), methyl (54), benzyl (98), 2-phenylethyl (101), propyl (63) and butyl (73) propanoate, which are correlated with the increased amount of propanoic acid (3) (Table 2). On the other hand, the HAL samples were mainly associated with acetate esters (ethyl (53), isobutyl (60), 3-methylbutyl (70), pentyl (74), hexyl (82) and (Z)-3-hexenyl (84) acetate), ethyl esters (ethyl octanoate (93), lactate (89), 3-hexenoate (83), hexanoate (80), pentanoate (72), 3-methylbutanoate (67), butanoate (62) and 2-methylpropanoate (56)) and methyl esters (methyl octanoate (91), lactate (85) and hexanoate (75)). The higher content of esters correlated positively to those of alcohols (ethanol (12), 2-methyl-1-propanol (15), 2- and 3-methyl-1-butanol (20, 21), 1-hexanol (26), (Z)-3-hexenol (27), 2,3-butanediol (35) and acids (acetic (1), hexanoic (9) and octanoic (10) acid). The differentiation of HLK tables olives according to geographical origin has been also confirmed by hierarchical cluster analysis (Figure S6). 

**Figure 5:**
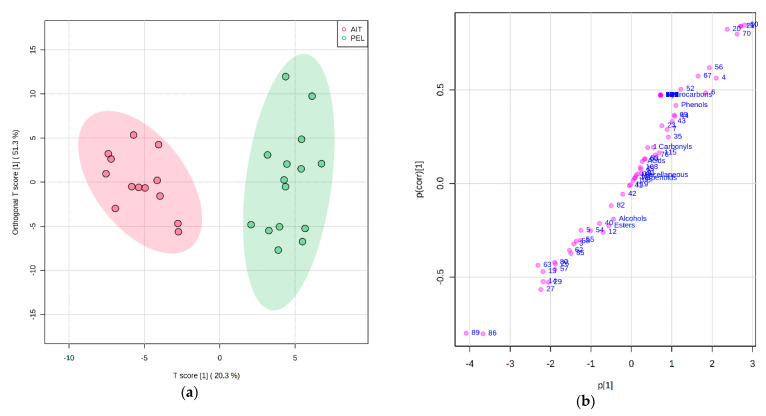
OPLS-DA of the volatile compounds identified in cv. Kalamata natural black olives grown in Aitoloakarnania (AIT—red dots) and southern Peloponnese (PEL—green dots): (**a**) scores plot, and (**b**) loadings S-plot. The shaded areas represent the 95% confidence ellipses. The codes of variables are given in Table 2.

KLM table olives were produced in Aitoloakarnania (AIT) and southern Peloponnese (PEL) (Figure S1) using the Greek-style method. Performing PCA resulted in unsatisfactory separation according to producing area, as there was partial overlap of the clusters (Figure S7). For this reason, orthogonal partial least-squares discriminant analysis (OPLS-DA) was performed, which resulted in clear discrimination between the AIT and PEL samples (Figure 5a). This mainly was due to the higher content of 3-methylbutyl acetate (70), isobutyl acetate (60), 2-methyl-1-butanol (20) and 3-methyl-1-butanol (21) in PEL samples, as well as to ethyl lactate (89) and propyl hexanoate (86) in AIT samples (Figure 5b). 

## 4. Conclusions

The results obtained from this study reveal the complex volatile profile of industrially fermented Halkidiki green olives, Conservolea and Kalamata natural black olives originating from different growing locations in Greece. More than 100 volatile compounds were identified in the pulp of olives and distributed over different chemical classes. All samples were rich in esters, alcohols and acids, whereas the samples of cv. Halkidiki were also rich in volatile phenols. Both qualitative and quantitative differences were observed between the olives produced from different cultivar as well as growing location. Based on multivariate analysis results, the samples were clearly separated according to olive cultivar and growing location. The table olives of the three cultivars were discriminated mostly due to the higher content of *trans*-β-ocimene and ethanol in cv. Kalamata, α-muurolene and α-farnesene in cv. Conservolea and guaiacol and 4-methylguaiacol in cv. Halkidiki samples. Within the olives of cv. Conservolea, those grown in Magnesia were clearly discriminated from the other two growing locations (Fthiotida, N. Evia) due to their high content in butanoic acid, butanol, butanoate esters and volatile phenols (mainly guaiacol). Within the olives of cv. Halkidiki, those grown in Kavala were richer in volatile phenols, propanoate esters and propanoic acid, whereas the samples from the Halkidiki region were mainly associated with acetate, ethyl and methyl esters. Although fewer differences were observed between the cv. Kalamata olives from Aitoloakarnania and S. Peloponnese, there was a clear separation due to the higher content of 3-methylbutyl acetate, isobutyl acetate, methyl acetate and 3-methyl-1-butanol in S. Peloponnese samples, as well as to ethyl lactate and propyl hexanoate in Aitoloakarnania samples. To the best our knowledge, this is the first systematic study on the volatile composition of industrially fermented table olives from Greek cultivars, highlighting the pronounced effect of growing location. However, to confirm our results, further studies should be undertaken using a higher number of samples spanning different harvesting periods. Finally, the link between volatile compounds and microbial composition of table olives should be established that would enable the design of strategies to improve quality and enhance the regional character of the final product. 

## Figures and Tables

**Table 1:** Coding and geographical origin of the fermented cv. Kalamata, cv. Conservolea and cv. Halkidiki olive samples.

Sample Code ^1^	Sample Number	Cultivar	Growing Area	Fermentation Type	Olive Color
KLM_AIT	14	Kalamata	Aitoloakarnania	Greek-style	Black
KLM_PEL	15		Southern Peloponnese		
CNS_FTH	6	Conservolea	Fthiotida		
CNS_MAG	6		Magnesia		
CNS_EVIA	3		Northern Evia		
HLK_HAL	9	Halkidiki	Halkidiki	Spanish-style	Green
HLK_KAV	6		Kavala		

^1^ The letters before the dash denote cultivar; the letters after the dash denote growing area.

**Table 2:** Volatile composition of Halkidiki, Conservolea and Kalamata table olives as affected by growing area.

		Content (% of Total Area of Identified Compounds) ^1^									
		Halkidiki Cultivar			Conservolea Cultivar				Kalamata Cultivar		
Code	Compound	HAL	KAV		FTH	EVIA	MAG		AIT	PEL	
*Acids*											
1	Acetic acid	18.06 a	12.02 b	A	8.67 a	11.33 b	7.57 a	B	15.56	16.98	A
2	Formic acid	0.17 a	0.05 b	A	0.04	0.12	0.03	A	nd	nd	B
3	Propanoic acid	3.29 a	17.84 b	A	3.34 a	4.10 b	4.10 b	B	3.38	2.39	B
4	2-Methylpropanoic acid	0.22	0.11		0.10 a	0.12 a	0.22 b		0.15 a	0.70 b	
5	Butanoic acid	0.34	0.35	A	0.33 a	0.22 a	19.33 b	B	0.33	1.55	A
6	3-Methylbutanoic acid	0.70	0.49	A	0.14 a	0.20 a	0.52 b	AB	0.08	0.28	B
7	2-Methylbutanoic acid	0.21	0.18		0.16 a	0.17 a	0.22 b		0.13	0.26	
8	Pentanoic acid	0.03	0.04	A	0.03 a	0.02 a	0.14 b	B	tr	tr	C
9	Hexanoic acid	0.36 a	0.13 b	A	0.22 a	0.10 b	0.22 a	B	0.03	0.04	C
10	Octanoic acid	0.15 a	0.03 b	A	0.03 a	0.02 b	0.04 a	B	nd	nd	C
11	Nonanoic acid	0.06	0.04	A	0.02	0.03	0.05	A	nd	nd	B
*Alcohols*											
12	Ethanol	5.59 a	1.02 b	A	3.47 a	4.63 b	1.80 c	A	20.53	20.29	B
13	2-Butanol	1.22 a	5.61 b		7.54 a	8.89 b	0.92 c		11.89	4.29	
14	1-Propanol	1.29	1.53		1.89 a	2.89 b	0.38 c		1.31	0.48	
15	2-Methyl-1-propanol	0.04	tr	A	0.01	tr	0.02	A	0.03 a	0.10 b	B
16	3-Pentanol	0.32 a	0.07 b	A	0.09 a	0.12 ab	0.14 b	B	0.02	0.02	C
17	2-Pentanol	0.22 a	0.09 b	A	0.04 a	0.05 a	0.16 b	B	0.02	0.02	C
18	1-Butanol	0.06	0.09	A	0.06 a	0.04 a	1.04 b	B	0.06	0.09	A
19	1-Penten-3-ol	0.02	tr	A	0.02 a	0.02 a	0.08 b	B	tr	tr	A
20	2-Methyl-1-butanol	1.49 a	0.16 b	A	0.55 a	0.26 a	0.77 b	A	0.94 a	2.54 b	B
21	3-Methyl-1-butanol	4.81 a	0.36 b	AB	1.13 a	0.65 a	2.33 b	A	2.20 a	7.57 b	B
22	3-Methyl-3-buten-1-ol	0.10 a	0.01 b	A	0.03 a	0.05 b	0.04 b	B	tr	tr	C
23	1-Pentanol	0.05 a	0.02 b	A	0.03 a	0.06 a	0.12 b	AB	0.07	0.08	B
24	3-Methyl-2-buten-1-ol	0.10 a	0.01 b	A	0.03 a	0.06 b	0.05 b	A	tr	tr	B
25	2-Heptanol	0.12 a	0.06 b	A	0.03 a	0.03 a	0.11 b	B	0.01	0.01	C
26	1-Hexanol	1.06 a	0.06 b	A	1.22 a	2.00 b	1.04 a	B	0.38	0.26	C
27	(Z)-3-Hexen-1-ol	1.60 a	0.12 b	A	1.12	1.16	1.15	A	0.39	0.18	B
28	3-Octanol	0.03 a	0.02 b	A	0.05	0.05	0.05	B	nd	nd	C
29	(E)-2-Hexen-1-ol	0.07 a	0.20 b	A	0.02 a	0.08 b	0.03 a	B	0.07	0.02	B
30	(Z)-2-Hexen-1-ol	0.02 a	0.06 b	A	0.04 a	0.07 b	tr a	A	nd	nd	B
31	1-Octen-3-ol	0.07 a	0.04 b	A	0.05 a	0.06 a	0.21 b	B	tr	0.01	C
32	1-Heptanol	0.22 a	0.02 b	A	0.15	0.14	0.16	A	tr	tr	B
33	2-Ethyl-1-hexanol	0.12	0.09	A	0.06 a	0.05 a	0.14 b	A	0.01	0.01	B
34	(E)-2-Hepten-1-ol	0.04	tr	A	0.03 a	0.18 b	0.02 a	A	nd	nd	B
35	2,3-Butanediol	2.67 a	0.32 b	A	0.35	0.33	0.24	B	0.20	0.56	B
36	1-Octanol	0.28 a	0.06 b	A	0.09 a	0.10 a	0.17 b	B	0.01	0.01	C
37	(E)-2-Octen-1-ol	0.02	0.04	A	0.02 a	0.04 b	0.01 a	A	nd	nd	B
38	1-Nonanol	0.09 a	0.04 b	A	0.16 a	0.17 a	0.11 b	B	nd	nd	C
39	1,3-Propanediol	0.08 a	0.13 b	A	0.20 a	0.10 ab	0.02 b	A	nd	nd	B
40	Benzyl alcohol	0.45 a	0.36 b	A	1.29 a	0.73 b	0.69 b	B	0.28	0.31	A
41	Phenylethyl Alcohol	3.99 a	1.98 b	A	2.17 a	2.42 ab	3.00 b	A	0.31	0.65	B
*Carbonyls*											
42	2-Butanone	0.12 a	0.69 b		0.24 a	0.27 a	0.47 b		0.27	0.19	
43	2-Methylbutanal	0.06 a	0.04 b	AB	0.03 a	0.03 a	0.11 b	A	0.03	0.03	B
44	3-Methylbutanal	0.09 a	0.04 b	A	0.04 a	0.04 a	0.14 b	A	0.29	0.42	B
45	Hexanal	0.02	0.03	A	0.04 a	0.04 a	0.07 b	AB	0.07	0.05	B
46	Acetoin	0.07	tr	A	tr a	tr a	0.03 b	B	tr	tr	B
47	6-Methyl-5-hepten-2-one	0.05 a	0.04 b	A	tr a	0.01 a	0.05 b	B	nd	nd	C
48	Nonanal	0.01	tr	A	0.01 a	0.04 b	0.02 c	B	tr	tr	C
49	Benzaldehyde	0.05	0.08	A	0.39 a	0.07 b	0.06 b	B	0.01	0.01	A
50	Phenylacetaldehyde	0.02	0.01	A	0.02 a	0.01 a	0.04 b	B	tr	tr	C
51	(E)-2-Decenal	0.08 a	0.01 b	A	0.03 a	0.06 b	0.07 b	A	nd	nd	B
*Esters*											
52	Methyl acetate	1.27	1.29	A	1.19 a	0.67 a	2.05 b	A	2.35 a	4.10 b	B
53	Ethyl Acetate	7.21 a	0.66 b	A	5.64 a	4.38 ab	3.74 b	A	7.38	9.56	B
54	Methyl propanoate	0.28 a	5.27 b	A	0.57 a	0.40 a	1.37 b	B	0.40	0.48	B
55	Ethyl propanoate	5.81	5.58	A	11.28	9.91	12.54	B	6.94	7.19	A
56	Ethyl 2-methylpropanoate	0.04	tr		0.04 a	0.06 b	0.02 c		0.03 a	0.14 b	
57	Propyl acetate	9.84 a	5.35 b	A	16.14 a	16.05 a	2.02 b	A	3.47	1.48	B
58	Methyl butanoate	0.02	0.01	A	0.02 a	0.01 a	1.21 b	B	0.05	0.11	AB
59	1-Methylpropyl acetate	0.03 a	0.34 b	A	0.71 a	0.50 b	0.38 c	B	tr	tr	C
60	Isobutyl acetate	0.82 a	0.06 b		0.33 a	0.17 b	0.30 a		0.15 a	0.62 b	
61	Methyl 3-methylbutanoate	0.06 a	0.03 b	A	0.01 a	0.02 a	0.03 b	B	tr	0.01	C
62	Ethyl butanoate	0.12 a	0.03 b		0.18 a	0.14 a	2.37 b		0.39	0.33	
63	Propyl propanoate	2.02 a	11.13 b	A	9.23 a	8.46 a	2.31 b	A	1.37	0.43	B
64	1-Methylpropyl propanoate	tr a	0.91 b	A	0.16 a	0.09 b	0.01 c	B	tr	tr	B
65	Ethyl 2-methylbutanoate	0.03	0.05	A	0.11 a	0.17 b	0.02 c	A	0.11	0.18	B
66	Propyl 2-methylpropanoate	tr	tr		0.01	0.02	tr		nd	nd	
67	Ethyl 3-methylbutanoate	0.10 a	0.02 b		0.06 a	0.11 b	0.03 c		0.06	0.15	
68	Butyl acetate	0.07	0.08	A	0.10 a	0.05 a	0.44 b	B	tr	tr	
69	2-Methylpropyl propanoate	0.15	0.31	A	0.23 a	0.11 b	0.30 c	A	0.08	0.15	B
70	3-Methylbutyl acetate	4.60 a	0.55 b		2.25 a	0.75 b	1.52 c		1.08 a	4.47 b	
71	Propyl butanoate	0.01	0.02	A	0.06 a	0.04 a	0.14 b	B	nd	nd	
72	Ethyl pentanoate	0.01	tr	A	0.03	0.02	0.02	B	nd	nd	
73	Butyl propanoate	0.01 a	0.29 b	A	0.08 a	0.03 a	0.71 b	B	tr	tr	C
74	Pentyl acetate	0.04 a	0.01 b	A	0.06 a	0.07 a	0.04 b	B	tr	tr	C
75	Methyl hexanoate	0.18 a	0.05 b	A	0.11 a	0.07 b	0.04 c	B	tr	tr	C
76	3-Methylbutyl propanoate	0.45	1.89	A	0.79 a	0.22 b	0.73 a	AB	0.21	0.43	B
77	3-Methyl-3-butenyl acetate	0.05	0.02	A	0.04	0.03	0.05	A	nd	nd	B
78	Butyl butanoate	nd	nd	A	nd a	nd a	0.85 b	B	tr	tr	A
79	Ethyl 3-methyl-2-butenoate	nd	nd	A	0.04 a	0.07 b	0.01 c	B	tr	tr	A
80	Ethyl hexanoate	0.48 a	0.02 b		0.36 a	0.24 b	0.04 c		0.20	0.19	
81	3-Methylbutyl butanoate	nd	nd	A	tr a	tr a	0.13 b	B	tr	tr	A
82	Hexyl acetate	0.28 a	0.03 b	A	0.60 a	0.54 a	0.17 b	B	0.08	0.08	A
83	Ethyl (E)-3-hexenoate	0.04 a	0.01 b	A	0.05 a	0.03 b	0.01 c	A	nd	nd	B
84	(Z)-3-Hexenyl acetate	0.54 a	0.05 b	A	0.76 a	0.41 b	0.23 c	A	tr	tr	B
85	Methyl lactate	0.36 a	0.02 b	A	0.15 a	0.26 b	0.03 c	B	0.05	0.03	B
86	Propyl hexanoate	tr	tr	A	0.04	0.03	tr	A	0.91 a	0.11 b	B
87	Ethyl heptanoate	0.01	nd	A	0.05	0.03	tr	B	nd	nd	C
88	Hexyl propanoate	0.01	0.03	A	0.12 a	0.08 b	0.05 b	B	tr	tr	C
89	Ethyl lactate	3.92 a	0.12 b	A	1.83 a	3.31 b	0.19 c	B	7.26 a	0.55 b	C
90	(Z)-3-Hexenyl propanoate	0.03 a	0.08 b	A	0.14 a	0.06 b	0.08 b	B	nd	nd	C
91	Methyl octanoate	0.23 a	0.02 b	A	0.04 a	0.02 b	0.02 b	B	0.01	0.09	B
92	Isopropyl lactate	0.27	0.13	A	0.71 a	1.07 b	0.05 c	B	0.01	0.01	C
93	Ethyl octanoate	0.46 a	tr b	A	0.12 a	0.06 b	0.01 c	B	tr	tr	B
94	Ethyl 2-hydroxy-4-methylpentanoate	0.06	tr	A	0.03 a	0.03 a	tr b	B	nd	nd	C
95	Methyl benzoate	tr	tr	A	0.03 a	0.01 b	tr b	B	nd	nd	C
96	Benzyl acetate	0.08	0.06	A	0.36 a	0.10 b	0.06 b	B	tr	tr	C
97	Methyl salicylate	0.03	0.03	A	0.18 a	0.14 b	0.03 c	B	nd	nd	A
98	Benzyl propanoate	tr a	0.11 b	A	0.05 a	0.01 b	0.01 b	A	nd	nd	B
99	Ethyl salicylate	tr	0.03	A	0.41 a	0.16 b	0.09 b	B	nd	nd	A
100	2-Phenylethyl acetate	0.52 a	0.29 b	A	0.48 a	0.26 b	0.21 b	B	0.03	0.03	C
101	2-Phenylethyl propanoate	0.08 a	0.55 b	A	0.09 a	0.06 b	0.06 b	B	0.01	0.01	B
	*Hydrocarbons*										
102	Octane	1.00 a	0.64 b	A	1.32 a	1.98 ab	2.14 b	B	0.12	0.13	C
103	Decane	0.01	0.02	A	0.04	0.02	0.04	B	tr	tr	C
104	1-Dodecene	0.04	0.04	A	1.22	1.07	1.44	B	tr	tr	A
105	(E)-4,8-Dimethylnona-1,3,7-triene	0.04 a	0.01 b	A	0.03	0.02	0.03	A	tr	tr	B
*Miscellaneous compounds*											
106	Dimethyl sulfide	0.41	0.44	A	1.64 a	1.66 a	2.99 b	B	1.66	1.56	B
107	2,5-Dimethylfuran	0.02	tr	A	tr a	0.01 a	0.06 b	B	tr	tr	C
108	2-Pentylfuran	0.02	0.02	A	0.07	0.10	0.08	A	0.17	0.15	B
109	Butyrolactone	0.02	0.02	A	0.02 a	0.04 b	0.03 c	B	nd	nd	C
110	γ-Hexalactone	tr	tr	A	0.05 a	0.04 b	0.02 c	B	nd	nd	C
111	δ-Octalactone	nd	nd	A	0.04 a	0.05 a	0.01 b	B	nd	nd	A
112	γ-Nonalactone	0.01	0.01	A	0.03	0.04	0.03	B	nd	nd	C
*Phenols*											
113	Guaiacol	tr a	6.42 b	A	0.02 a	0.02 a	3.03 b	AB	tr	tr	B
114	4-Methylguaiacol	7.12 a	9.68 b	A	1.62 a	1.28 a	2.12 b	B	0.42	0.45	C
115	Phenol	0.02 a	1.08 b		0.02 a	0.04 a	1.01 b		0.51	0.97	
116	4-Ethylguaiacol	tr	0.02	A	0.02 a	tr b	0.06 c	B	nd	nd	C
117	4-Methylphenol	0.05 a	0.10 b	A	0.05	0.03	0.05	B	nd	nd	C
118	4-Ethylphenol	0.03 a	0.90 b	A	0.25 a	0.07 a	1.42 b	A	nd	nd	B
*Terpenoids*											
119	Limonene	0.05	0.10		0.06 a	0.08 a	0.13 b		0.12	0.06	
120	trans-β-Ocimene	0.01	0.01	A	0.50	0.44	0.51	A	5.10	5.15	B
121	Rose oxide	0.01 a	0.04 b	A	tr	tr	0.01	B	nd	nd	C
122	Copaene	0.03	0.02	A	0.06 a	0.13 a	0.48 b	B	nd	nd	A
123	Linalool	0.02	0.02	A	0.02	0.01	0.02	A	nd	nd	B
124	α-Terpineol	0.02	0.01	A	0.02	0.02	0.02	A	nd	nd	B
125	α-Muurolene	nd	nd	A	0.01 a	0.03 a	0.09 b	B	nd	nd	A
126	α-Farnesene	nd	nd	A	0.88	0.87	0.93	B	nd	nd	A

^1^ Mean value (the number of samples is given in Table 1). Data in the same row with different lowercase letters are significantly different within each cultivar (*p* < 0.05). Different uppercase letters denote that the means between the cultivars are significantly different (*p* < 0.05); tr: < 0.01%; nd: not detected (missing values were replaced by the 1/5 of the minimum value of each compound). The codes of growing areas are given in Table 1.

## Data Availability

The data presented in this study are available on request from the corresponding author. The data are not publicly available due to privacy restrictions.

## Supplementary Materials

The following are available online at https://www.mdpi.com/article/10.3390/foods10051000/s1, Table S1: Volatile compounds identified in the headspace of table olives from Kalamata, Conservolea and Halkidiki cultivars. Figure S1: Geographical origin of cv. Kalamata, cv. Conservolea and cv. Halkidiki table olives. Figure S2: PCA loadings plots of the volatile compounds identified in industrially fermented table olives from different cultivars: (**a**) Loadings 1 *vs* Loadings 2; (**b**) Loadings 1 *vs* Loadings 3. Figure S3: Heatmap plot of hierarchical cluster analysis performed on volatile compounds of table olives from CNS (cv. Conservolea), HLK (cv. Halkidiki) and KLM (cv. Kalamata) cultivars. Sample codes are given in Table 1. Variables codes are given in Table 2. Figure S4: Heatmap plot of hierarchical cluster analysis performed on volatile compounds of cv. Conservolea table olives from Northern Evia (EVIA), Fthiotida (FTH) and Magnesia (MAG) growing locations. Variables codes are given in Table 2. Figure S5: Principal component analysis of the volatile compounds identified in cv. Halkidiki green olives grown in Halkidiki (HAL) and Kavala (KAV): (**a**) scores plot and (**b**) loadings plot of the first vs third principal component. The codes of variables are given in Table 2. Figure S6: Heatmap plot of hierarchical cluster analysis performed on volatile compounds of cv. Halkidiki table olives from Halkidiki (HAL) and Kavala (KAV) growing locations. Sample codes are given in Table 1. Variables codes are given in Table 2. Figure S7: Principal component analysis of the volatile compounds identified in cv. Kalamata natural black olives grown in Aitoloakarnania (AIT—red dots) and Southern Peloponnese (PEL—green dots). The shaded areas represent the 95% confidence ellipses. Figure S8: Influence plot of F-residuals vs. Hotelling’s T2 statistics for PCA presented in: (**a**) Figure 2, (**b**) Figure 3 and (**c**) Figure 4.
