# Supplementary material for: Volatile Composition of Industrially Fermented Table Olives from Greece
Source: Foods. 2021 May 2;10(5):1000. doi: 10.3390/foods10051000 (PMC8147446; doi:10.3390/foods10051000)
Supplement: Supplementary file 1 [file foods-10-01000-s001.zip › foods-1173503-supplementary.pdf]

Table S1. Volatile compounds identified in the headspace of table olives from Kalamata, Conservolea and Halkidiki cultivars

Code	Compound	RI [1]	TI (m/z) [2]	RID [3]
Acids				
1	Acetic acid	1448	60	A
2	Formic acid	1503	46	A
3	Propanoic acid	1538	74	A
4	2-Methylpropanoic acid	1570	43	B
5	Butanoic acid	1630	60	B
6	3-Methylbutanoic acid	1672	60	B
7	2-Methylbutanoic acid	1673	74	B
8	Pentanoic acid	1739	60	B
9	Hexanoic acid	1846	60	A
10	Octanoic acid	2065	60	A
11	Nonanoic acid	2175	60	B
Alcohols				
12	Ethanol	932	46	A
13	2-Butanol	1031	45	B
14	1-Propanol	1043	59	A
15	2-Methyl-1-propanol	1098	74	A
16	3-Pentanol	1114	59	A
17	2-Pentanol	1128	45	A
18	1-Butanol	1150	56	A
19	1-Penten-3-ol	1166	57	B
20	2-Methyl-1-butanol	1212	57	A
21	3-Methyl-1-butanol	1213	55	A
22	3-Methyl-3-buten-1-ol	1253	56	B
23	1-Pentanol	1256	55	A
24	3-Methyl-2-buten-1-ol	1326	71	B
25	2-Heptanol	1327	45	B
26	1-Hexanol	1359	56	A
27	(Z)-3-Hexenol	1388	67	B
28	3-Octanol	1400	59	B
29	(E)-2-Hexen-1-ol	1408	57	B
30	(Z)-2-Hexen-1-ol	1413	57	B
31	1-Octen-3-ol	1456	57	A
32	1-Heptanol	1462	56	B
33	2-Ethyl-1-hexanol	1495	57	B
34	(E)-2-Hepten-1-ol	1516	57	B
35	2,3-Butanediol	1546	45	A
36	1-Octanol	1564	56	A
37	(E)-2-Octen-1-ol	1619	57	B
38	1-Nonanol	1667	56	B
39	1,3-Propanediol	1785	57	B
40	Benzyl alcohol	1878	108	A
41	Phenylethyl Alcohol	1915	91	A
Carbonyls				
42	2-Butanone	900	72	A
43	2-Methylbutanal	909	58	B

Code	Compound	RI [1]	TI (m/z) [2]	RID [3]
44	3-Methylbutanal	912	44	B
45	Hexanal	1078	44	A
46	Acetoin	1282	45	B
47	6-Methyl-5-hepten-2-one	1337	108	B
48	Nonanal	1392	98	B
49	Benzaldehyde	1519	106	A
50	Phenylacetaldehyde	1641	91	B
51	(E)-2-Decenal	1645	55	B
Esters				
52	Methyl acetate	821	74	A
53	Ethyl Acetate	883	61	A
54	Methyl propanoate	905	88	A
55	Ethyl propanoate	949	57	A
56	Ethyl 2-methylpropanoate	958	71	A
57	Propyl acetate	968	43	A
58	Methyl butanoate	982	74	A
59	1-Methylpropyl acetate	984	87	B
60	Isobutyl acetate	1013	43	A
61	Methyl 3-methylbutanoate	1018	74	B
62	Ethyl butanoate	1035	71	A
63	Propyl propanoate	1044	57	B
64	1-Methylpropyl propanoate	1051	101	B
65	Ethyl 2-methylbutanoate	1051	102	B
66	Propyl 2-methylpropanoate	1053	71	B
67	Ethyl 3-methylbutanoate	1067	85	B
68	Butyl acetate	1071	56	A
69	2-Methylpropyl propanoate	1079	57	B
70	3-Methylbutyl acetate	1122	43	A
71	Propyl butanoate	1123	89	B
72	Ethyl pentanoate	1136	88	A
73	Butyl propanoate	1141	57	B
74	Pentyl acetate	1173	43	B
75	Methyl hexanoate	1186	74	A
76	3-Methylbutyl propanoate	1189	57	B
77	3-Methyl-3-butenyl acetate	1194	68	C
78	Butyl butanoate	1219	71	B
79	Ethyl 3-methyl-2-butenoate	1224	83	C
80	Ethyl hexanoate	1234	88	A
81	3-Methylbutyl butanoate	1267	71	B
82	Hexyl acetate	1273	43	A
83	Ethyl (E)-3-hexenoate	1301	69	B
84	(Z)-3-Hexenyl acetate	1317	67	B
85	Methyl lactate	1319	45	B
86	Propyl hexanoate	1320	117	B
87	Ethyl heptanoate	1334	88	A
88	Hexyl propanoate	1341	57	B
89	Ethyl lactate	1345	45	A
90	(Z)-3-Hexenyl propanoate	1385	57	B

Code	Compound	RI [1]	TI (m/z) [2]	RID [3]
91	Methyl octanoate	1390	74	A
92	Isopropyl lactate	1429	45	C
93	Ethyl octanoate	1437	88	A
94	Ethyl 2-hydroxy-4-methylpentanoate	1547	69	B
95	Methyl benzoate	1621	105	B
96	Benzyl acetate	1728	108	B
97	Methyl salicylate	1771	120	B
98	Benzyl propanoate	1791	108	B
99	Ethyl salicylate	1805	120	B
100	2-Phenylethyl acetate	1813	104	A
101	2-Phenylethyl propanoate	1886	104	C
Hydrocarbons				
102	Octane	797	85	A
103	Decane	1000	57	A
104	1-Dodecene	1242	55	B
105	(E)-4,8-Dimethylnona-1,3,7-triene	1306	69	C
Miscellaneous compounds				
106	Dimethyl sulfide	739	62	A
107	2,5-Dimethylfuran	944	96	B
108	2-Pentylfuran	1230	81	B
109	Butyrolactone	1624	86	B
110	γ-Hexalactone	1701	85	B
111	δ-Octalactone	1974	99	B
112	γ-Nonalactone	2032	85	C
Phenols				
113	Guaiacol	1859	109	A
114	4-Methylguaiacol	1957	123	B
115	Phenol	2006	94	A
116	4-Ethylguaiacol	2032	137	C
117	4-Methylphenol	2085	107	B
118	4-Ethylphenol	2182	107	B
Terpenoids				
119	Limonene	1191	93	A
120	trans-β-Ocimene	1250	93	B
121	Rose oxide	1352	139	B
122	Copaene	1489	119	B
123	Linalool	1553	93	A
124	α-Terpineol	1702	93	A
125	α-Muurolene	1724	105	B
126	α-Farnesene	1748	93	B

[1] Experimental retention index on DB-Wax column

[2] Target ion used to obtain peak area of each compound

[3] Reliability of identification: A, agreement of RI and MS spectra with those of an authentic compound analyzed under identical experimental conditions; B, agreement of RI (ΔRI<20) and MS (match>900); C, at least MS similarity match>800.

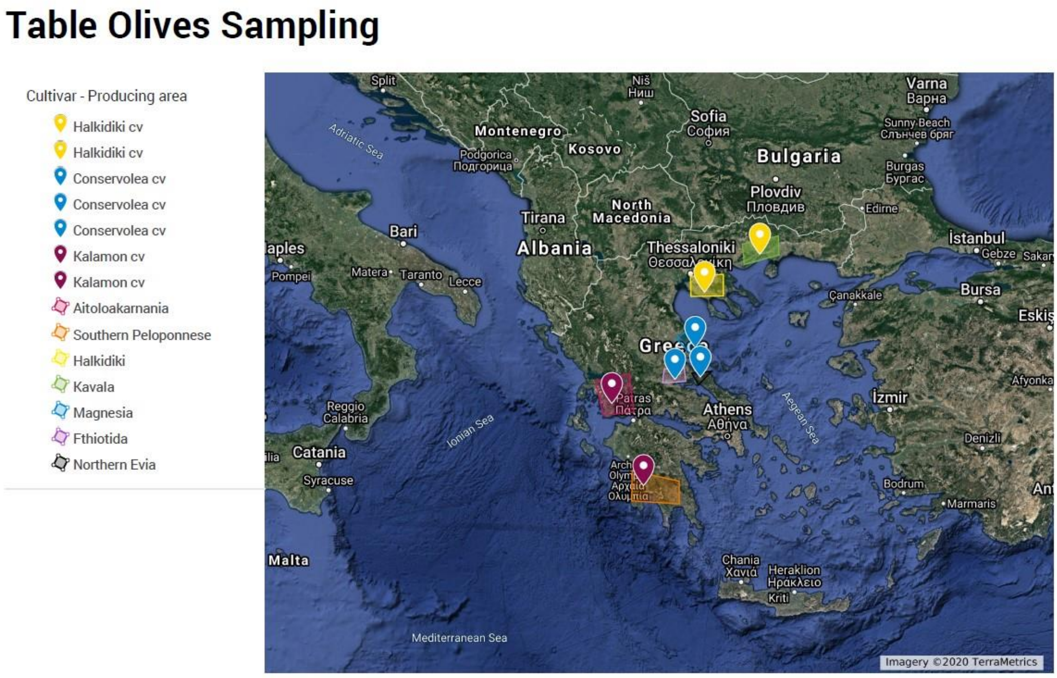

Figure S1. Geographical origin of cv. Kalamata, cv. Conservolea and cv. Halkidiki table olives.

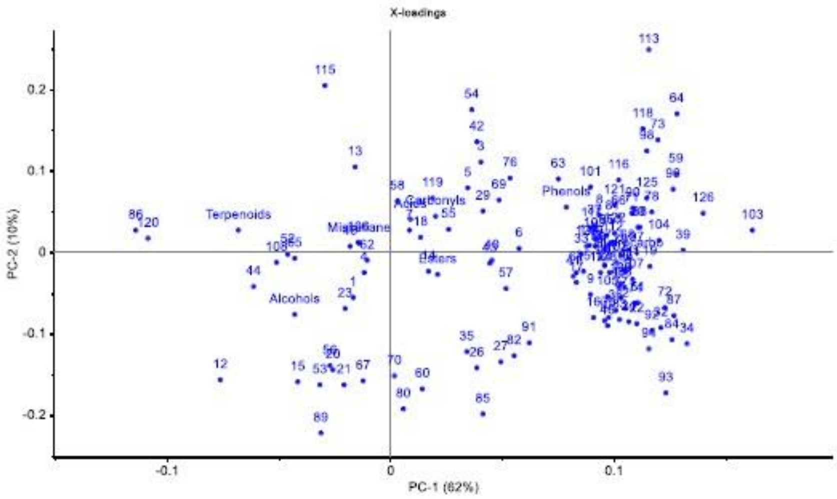

(a)

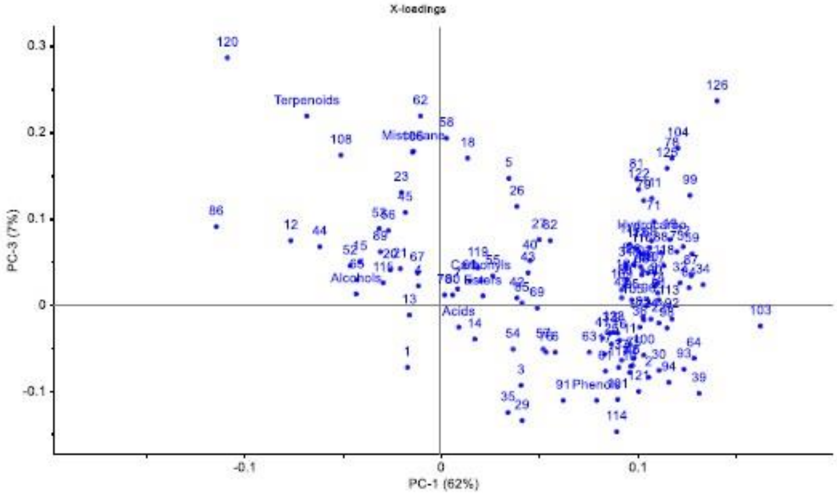

(b)

Figure S2. PCA loadings plots of the volatile compounds identified in industrially fermented table olives from different cultivars: (**a**) Loadings 1 vs Loadings 2; (**b**) Loadings 1 vs Loadings 3.

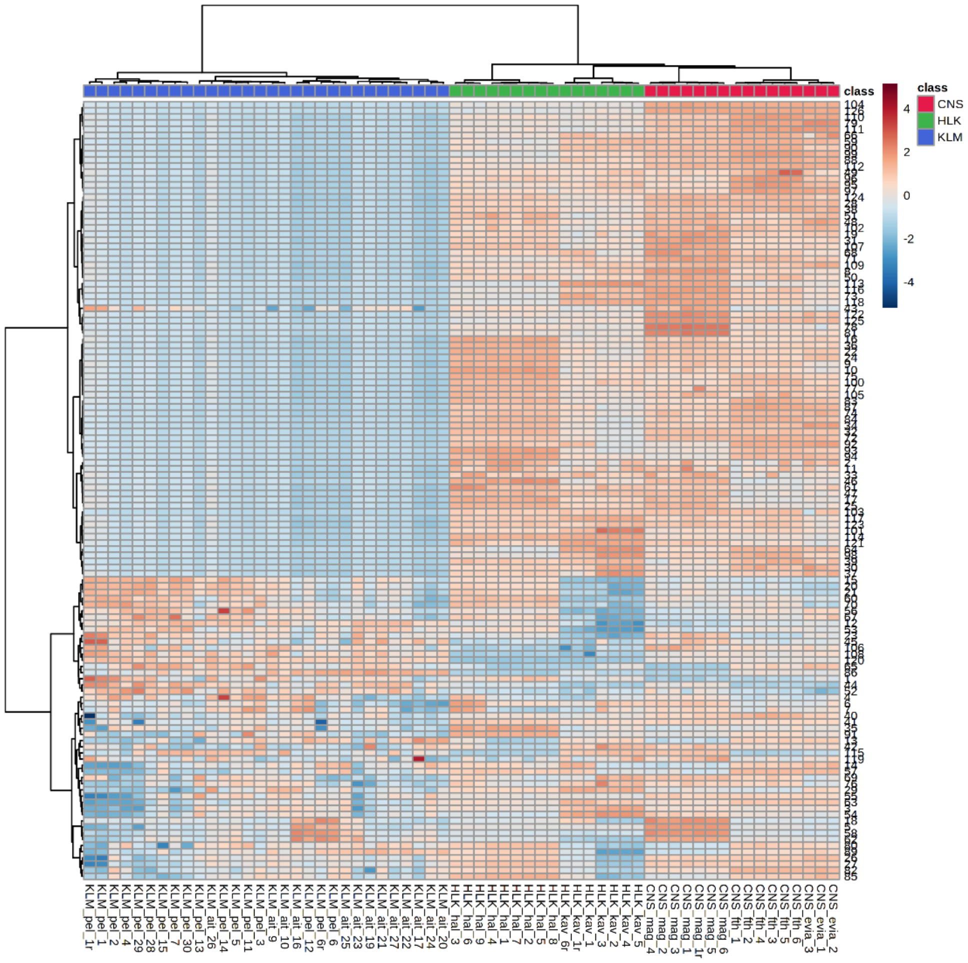

Figure S3: Heatmap plot of hierarchical cluster analysis performed on volatile compounds of table olives from CNS (cv. Conservolea), HLK (cv. Halkidiki) and KLM (cv. Kalamata) cultivars. Sample codes are given in Table 1. Variables codes are given in Table 2.

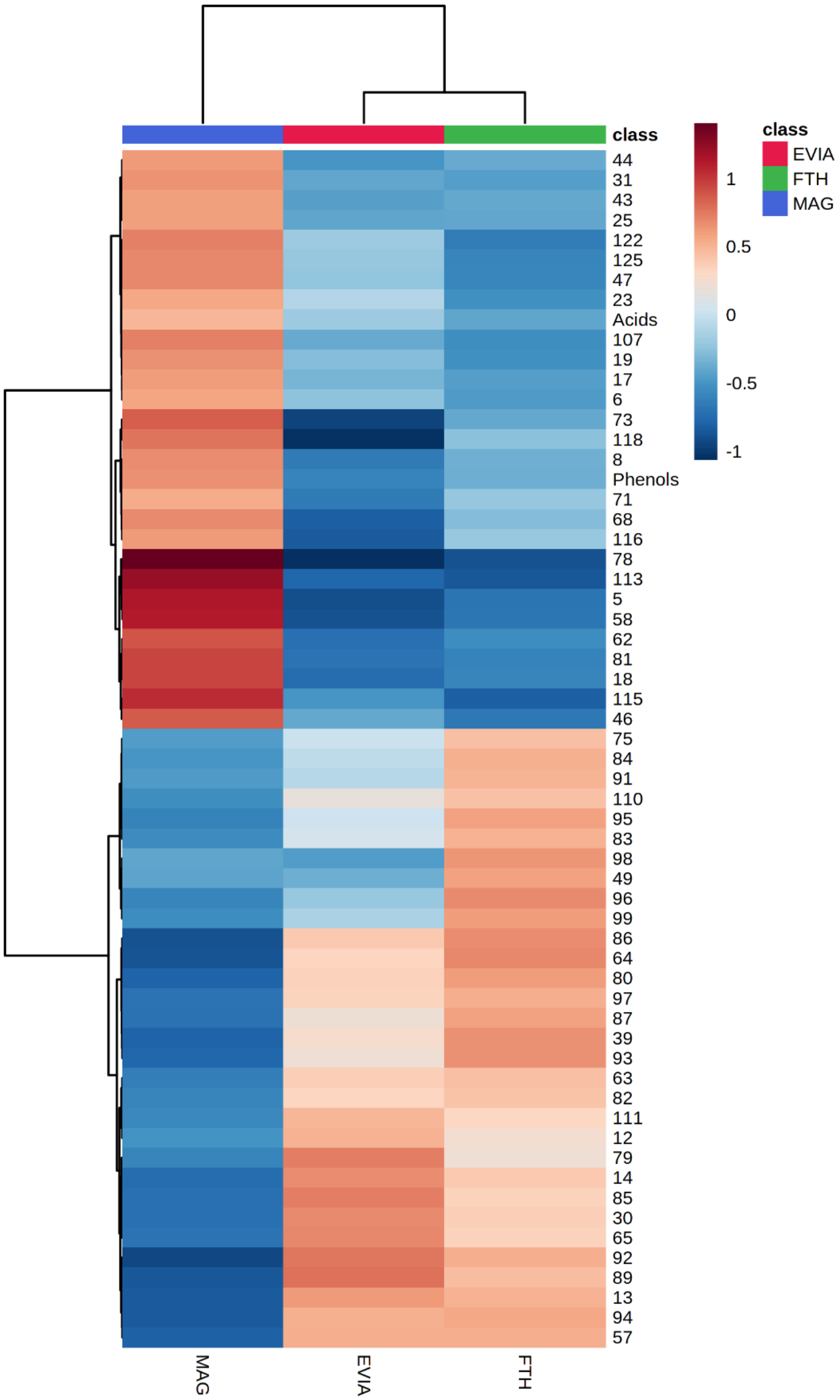

Figure S4: Heatmap plot of hierarchical cluster analysis performed on volatile compounds of cv. Conservolea table olives from Northern Evia (EVIA), Fthiotida (FTH) and Magnesia (MAG) growing locations. Variables codes are given in Table 2.

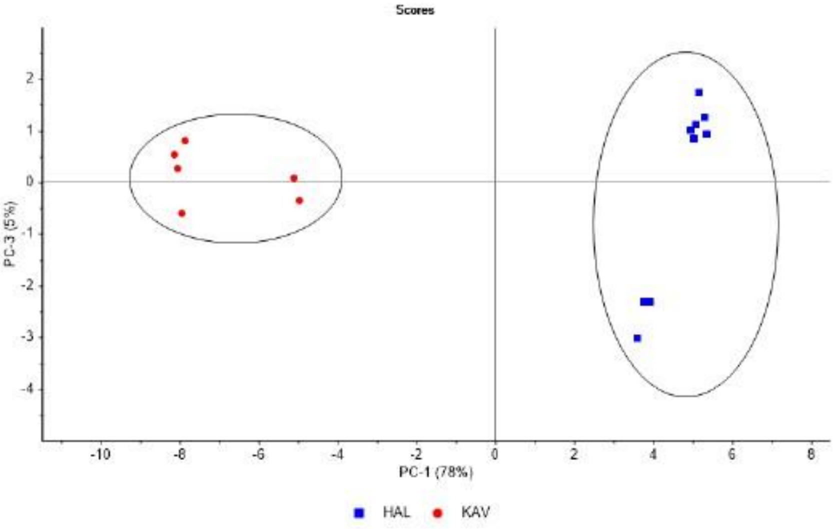

(a)

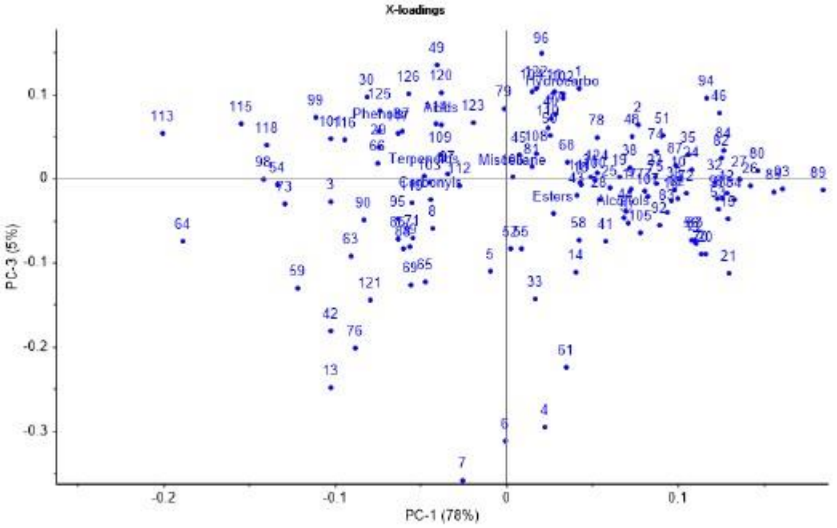

(b)

Figure S5: Principal component analysis of the volatile compounds identified in cv. Halkidiki green olives grown in Halkidiki (HAL) and Kavala (KAV): (a) scores plot and (b) loadings plot of the first vs third principal component. The codes of variables are given in Table 2.

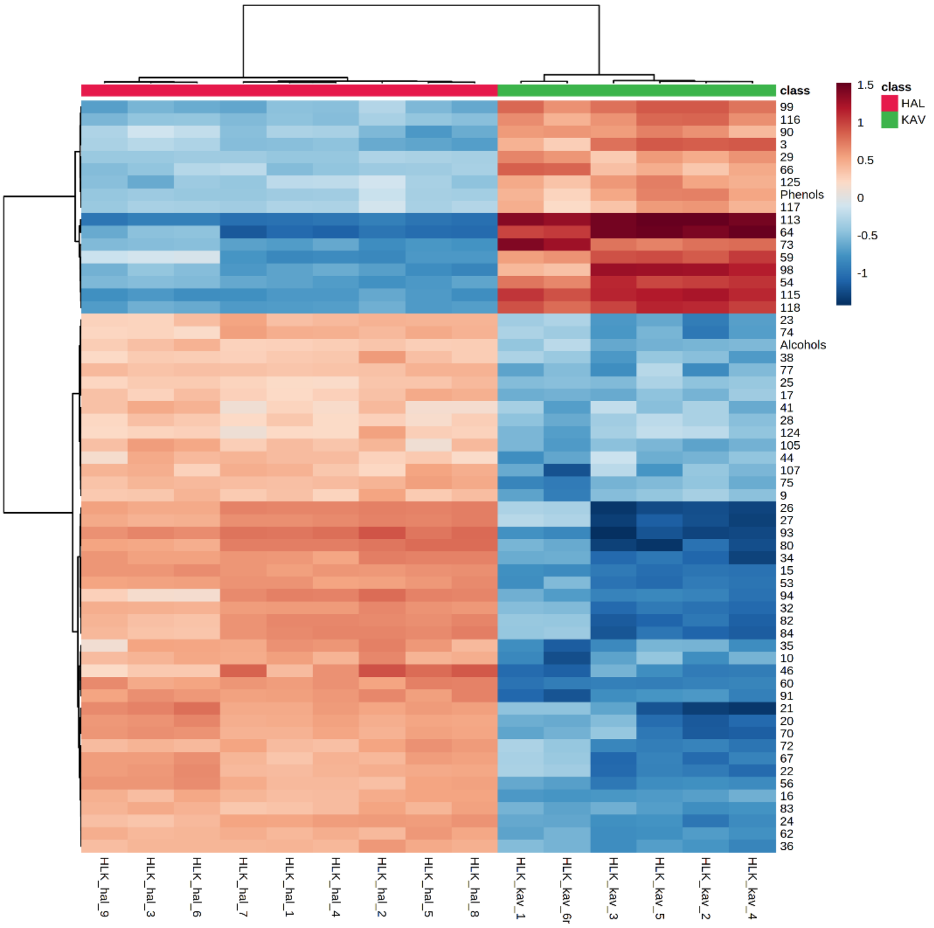

Figure S6: Heatmap plot of hierarchical cluster analysis performed on volatile compounds of cv. Halkidiki table olives from Halkidiki (HAL) and Kavala (KAV) growing locations. Sample codes are given in Table 1. Variables codes are given in Table 2.

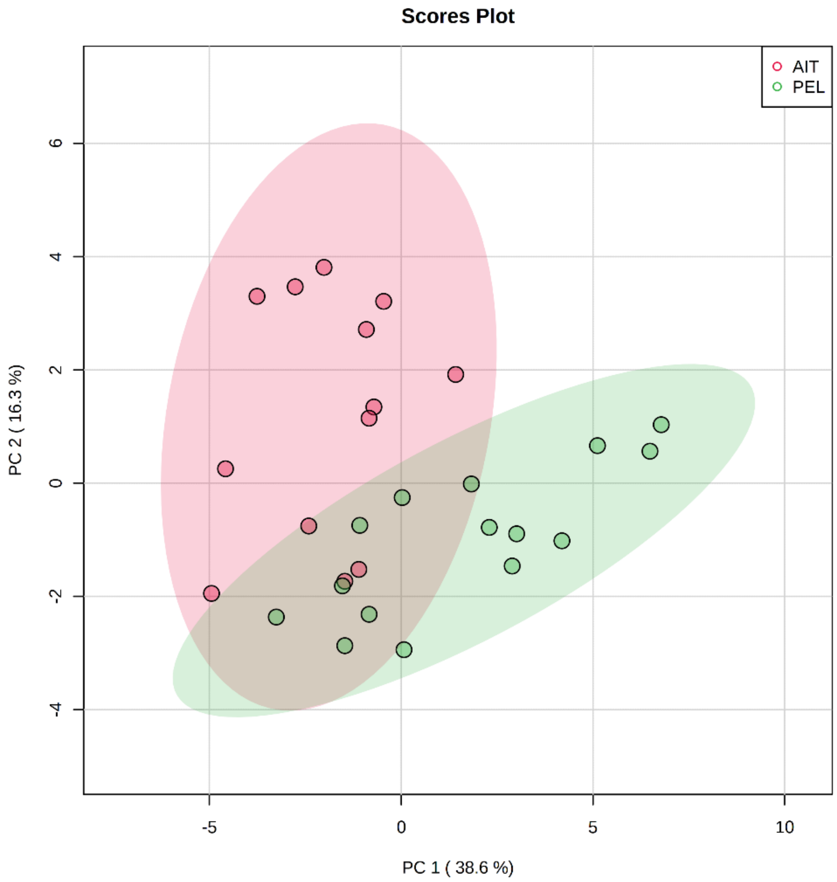

Figure S7: Principal component analysis of the volatile compounds identified in cv. Kalamata natural black olives grown in Aitoloakarnania (AIT – red dots) and Southern Peloponnese (PEL – green dots). The shaded areas represent the 95% confidence ellipses.

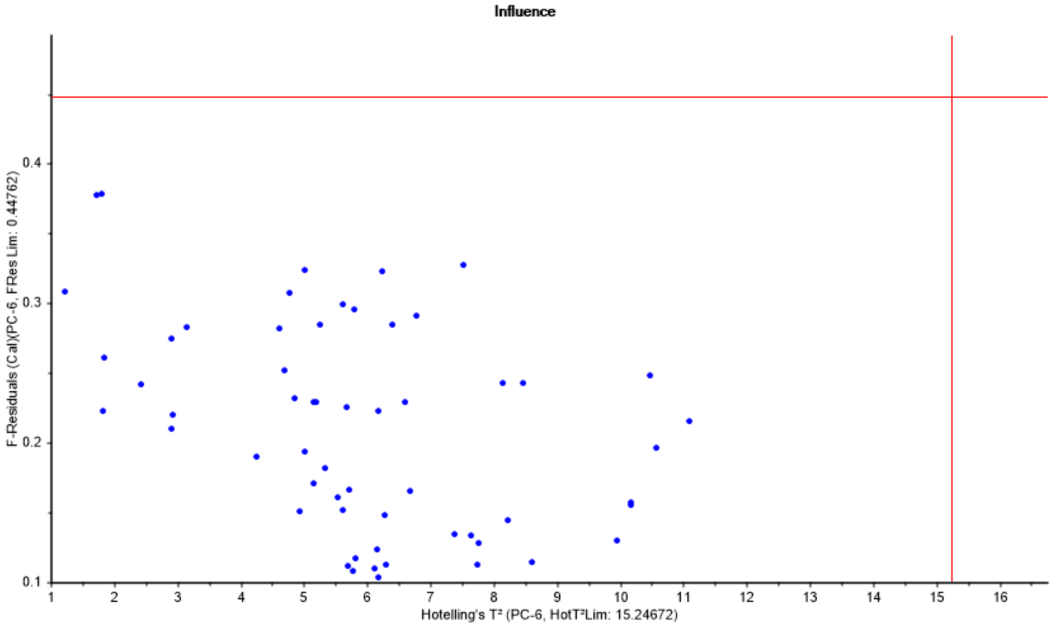

(a)

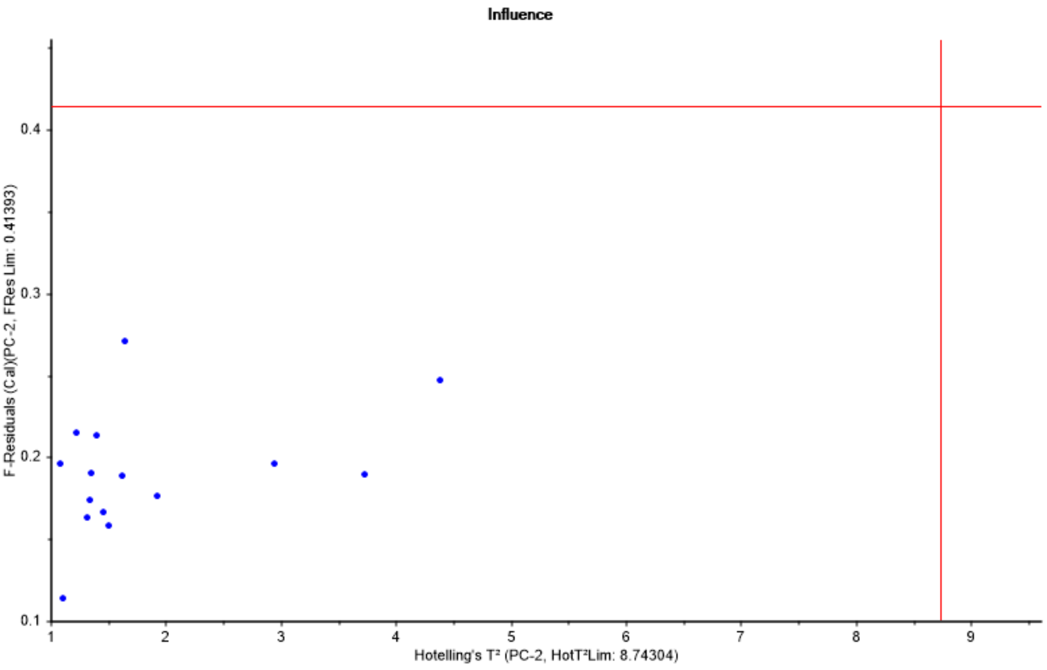

(b)

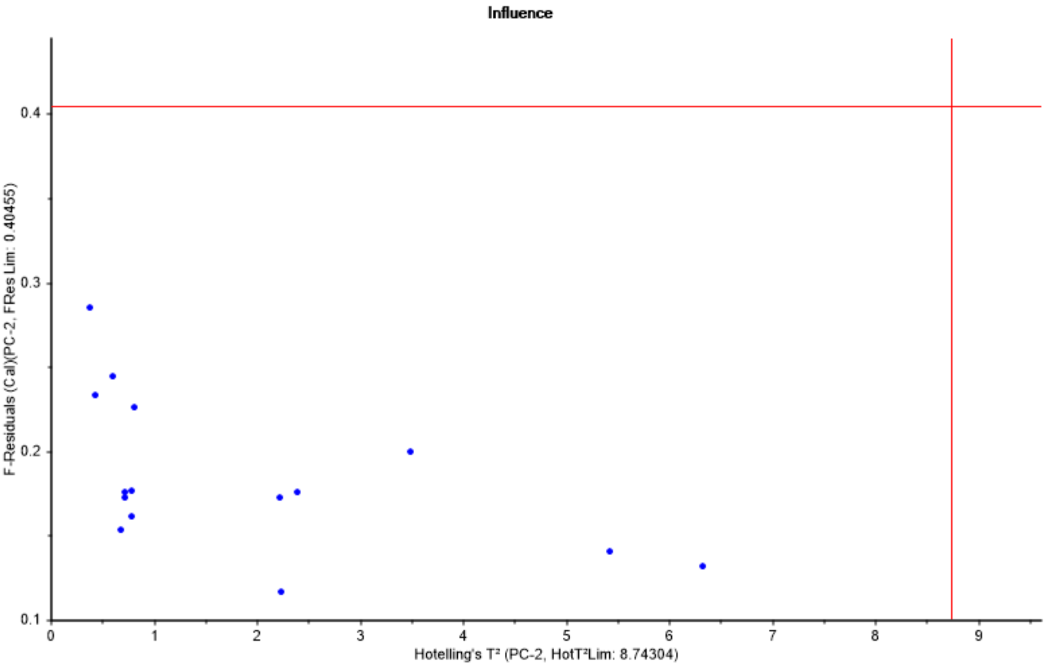

(c)

Figure S8: Influence plot of F-residuals vs. Hotelling's T2 statistics for PCA presented in: a) Figure 2, b) Figure 3 and c) Figure 4.
